# Cardiometabolic Diseases and Related Complications: Current Status and Future Perspective

**DOI:** 10.1155/2013/467682

**Published:** 2013-10-09

**Authors:** Joseph Fomusi Ndisang, Sharad Rastogi

**Affiliations:** ^1^Department of Physiology, College of Medicine, University of Saskatchewan, 107 Wiggins Road, Saskatoon, SK, Canada S7N 5E5; ^2^Division of Cardiovascular Medicine, Department of Medicine, Henry Ford Heart & Vascular Institute, 2799 West Grand Boulevard, Detroit, MI 48202, USA

In the past decade, there has been a dramatic increase in chronic diseases like diabetes, hypertension, and obesity not only in industrialized nations but also in developing nations with emerging economies [1, 2]. With the escalation of obesity, diabetes and hypertension, there has been a parallel increase in the incidence and prevalence of cardiometabolic complications [3, 4]. Cardiometabolic complications are multifactorial diseases, and a wide spectrum of different factors including changes in living environments, diets, lifestyles, genetic, and epigenetic factors [1, 2] may be involved. Although significant strides have been made in elucidating the multifaceted mechanisms associated with many cardiometabolic complications [1, 2], much still has to be done. For example studies which unveil novel mechanisms implicated in cardiometabolic diseases may open new therapeutic horizons.

This special issue focuses on original research articles and review papers that address a broad range of mechanisms associated with cardiometabolic diseases, with possible prognostic and therapeutic interventions. It is widely acknowledged that genetic defects are implicated in many cardiometabolic diseases including hypertension [5]. Genetic defects may be caused by altered exonic splicing leading to aberrant gene regulation. In an article featured in this special issue, L. Zhang and coworkers investigated the association between polymorphisms of *α*-adducin (ADD1) gene and essential hypertension. The authors reported that chromosome rs4963 within the ADD1 gene is associated with the development of essential hypertension in Chinese people, particularly males. Furthermore, the authors reported that the interaction among body mass index, chromosomes rs4963 and rs16843452 constitute an additional detrimental factor that increases the susceptibility to developing essential hypertension. Defective genes are also implicated in impaired lipid metabolism and lipotoxicity [6]. This concept is further elaborated in an article by Z. Song and coworkers that is also featured in this special issue.

Besides defective genes, increased inflammatory episodes are among the pathophysiological driving force in many cardiometabolic complications [7]. Proinflammatory cytokines such as resistin, tumour necrosis factor alpha (TNF-*α*), interleukin (IL)-6, and IL1*β* are widely acknowledged as important pathophysiological factors implicated in insulin resistance [7]. In a related study, the interaction between resistin and insulin signaling is elaborated in this special issue in a research article by Z. W. Du et al. The authors showed that human resistin inhibits myogenic differentiation and causes insulin resistance in myocytes. Similarly, P. C. Tsiotra and coworkers reported that the expression of resistin, TNF-*α*, IL-6, and IL1*β* in human mononuclear cells was enhanced by hyperinsulinemia and hyperleptinemia in the chronic conditions of obesity, type 2 diabetes, and atherosclerosis. The authors suggested a pathophysiological role of Proinflammatory cytokines in dysfunctional insulin signaling, impaired endothelial function, and dyslipidemia. If these detrimental factors are corrected in a timely manner, their progressive and ultimate transformation into more complicated cardiometabolic diseases may be avoided.

For optimal physiological functions adequate levels of vitamins are necessary. For example, vitamin D is known to enhance insulin sensitivity [8]. However, the effect of vitamin D on insulin sensitivity may be compromised in obese individuals [8]. Accordingly, the article by G. De Pergola et al. showed that in obese individuals, hyperinsulinemia and/or insulin resistance may be responsible for reducing the levels of vitamin D. In another related article featured in this issue, the pathophysiological role of atherogenic lipoproteins such as lipoprotein(a) was examined. Lipoprotein(a) is known to be critical in the development of many cardiovascular pathologies [9]. A detailed analysis of lipoprotein(a) can be found in the review article written by M. Malaguarnera et al. The authors examined the role of lipoprotein(a), in arteriosclerosis, coronary artery disease, and myocardial infarction. Many cardiometabolic complications including hyperlipidemia, dyslipidemia, obesity, type 2 diabetes, hypertension, nephropathy, nonalcoholic fatty liver disease are closely interrelated [7, 10]. Y. Li et al. wrote an article that is included in this special issue that underscores this paradigm.

Besides dyslipidemia, metabolic syndrome and several cardiovascular complications are associated with excessive production of glucocorticoids like cortisol [11]. C. G. Schnackenberg and coworkers expanded on this theme with an article for this issue that elaborates the effects of blocking 11*β*-hydroxysteroid dehydrogenase type 1 (11*β*-HSD1), a key enzyme necessary for the conversion of cortisone to cortisol on metabolic syndrome and cardiometabolic complications. The authors reported that blockade 11*β*-HSD1 in the liver and adipose tissue leads to reduction of mean arterial pressure, glucose intolerance, insulin resistance, hypertriglyceridemia, and plasma renin activity with no effect on heart rate, body weight gain, or microalbuminuria. It was suggested that 11*β*-HSD1 may be common mediator of hypertension, hypertriglyceridemia, glucose intolerance, and insulin resistance in metabolic syndrome. Thus, novel pharmaceutical agents capable of lowering lipoprotein(a) and blocking cortisol may retard or suppress the development of many cardiometabolic complications. Accordingly, the study of P. Aramwit and coworkers in this issue proposed a substance which could be explored in the search for novel remedies capable to suppress triglyceride, low-density lipoprotein, and C-reactive protein in patients with dyslipidemia. Similarly, the article by C. Liu and coauthors suggested the possible application of a substance known as the early growth response gene-1 DNA enzyme (EDRz) against cardiovascular complications like intimal hyperplasia and excessive proliferation of vascular smooth muscle cells. Agents that modulate EDRz may have pharmacological application.

Many physiological responses are impaired in patients with cardiometabolic disease and other chronic or debilitating conditions. For example, respiratory insufficiency is common in patients with cardiovascular complications. This notion has been discussed in detail in another article featured in this special issue by M. G. Neto et al. Similarly, response to ischemic insults may also be impaired in cardiovascular diseases. In a related study in this special issue, R. Schier et al. showed that patients with cardiovascular risk and particularly with those hypertension and diabetes mellitus had an aberrant reactive hyperemic response to ischemic insults, and, interestingly, this defect was improved by exercise. Moreover, ischemic insults are implicated in myocardial infarction [7, 12]. To expand on this topic, the article by H. Doods and D. Wu showed that sabiporide, a selective inhibitor of Na^+^/H^+^ exchanger, reduced ischemic insult in the myocardium and attenuated the severity of ventricular arrhythmias and myocardial infarct size. The underlying mechanism for the cardioprotective effect of sabiporide was attributed in part to the inhibition of ERK1/2 phosphorylation and suppression of inducible nitric oxide.

Diabetes is among the major causes of cardiometabolic complications. A common problem in diabetic patients is the maintenance of glycemic levels within a fairly narrow range, reflecting the recommendations of the Diabetes Control and Complications Trial [13, 14]. Although such tight glycemic control may not be feasible, the study of P. Kopecký et al., in this issue, suggests that a combination approach of two methodologies such as enhanced model predictive control algorithm and continuous glucose monitoring would be more reliable and accurate. Similarly, the usage of two or more antihypertensive drugs is a common practice for the management of hypertension [15]. In this special issue C. Y. Huang et al. discussed polytherapy in the treatment and management of hypertension. 

Taken together, the manuscripts in this special issue are based on the recent developments and future perspectives of cardiometabolic diseases. Importantly, these articles also unmask many challenging issues that should be overcome to improve diagnosis, prognosis, treatment and management of cardio-metabolic diseases and related complications.

*Joseph Fomusi Ndisang* *Sharad Rastogi*
